# Investigation into the Phytochemical Composition, Antioxidant Properties, and In-Vitro Anti-Diabetic Efficacy of *Ulva lactuca* Extracts

**DOI:** 10.3390/md22060240

**Published:** 2024-05-25

**Authors:** Safae Ouahabi, Nour Elhouda Daoudi, El Hassania Loukili, Hbika Asmae, Mohammed Merzouki, Mohamed Bnouham, Allal Challioui, Belkheir Hammouti, Marie-Laure Fauconnier, Larbi Rhazi, Alicia Ayerdi Gotor, Flore Depeint, Mohammed Ramdani

**Affiliations:** 1 Laboratory of Applied and Environmental Chemistry (LCAE), Faculty of Sciences, Mohammed First University, B.P. 717, Oujda 60000, Morocco; ouahabi.safae@ump.ac.ma (S.O.); asmaae.hbika@gmail.com (H.A.); moh.merzouki@gmail.com (M.M.); allal.challioui@gmail.com (A.C.); moharamdani2000@yahoo.fr (M.R.); 2 Laboratory of Bioresources, Biotechnology, Ethnopharmacology and Health, Faculty of Sciences, Mohammed First University, B.P. 717, Oujda 60000, Morocco; nourelhoudada95@gmail.com (N.E.D.); mbnouham@ump.ac.ma (M.B.); 3 Higher Institute of Nursing Professions and Health Techniques, Oujda 60000, Morocco; 4 Euromed Research Center, Euromed Polytechnic School, Euromed University of Fes (UEMF), Fes 30000, Morocco; e.loukili@ump.ac.ma (E.H.L.); hammoutib@gmail.com (B.H.); 5 Laboratory of Chemistry of Natural Molecules, University of Liège, Gembloux Agro-Bio Tech. 2, Passage des Déportés, B-5030 Gembloux, Belgium; marie-laure.fauconnier@uliege.be; 6 Institut Polytechnique UniLaSalle, Université d’Artois, ULR 7519, UniLaSalle, 19 rue Pierre Waguet, BP 30313, 60026 Beauvais, France; flore.depeint@unilasalle.fr; 7 Institut Polytechnique UniLaSalle, AGHYLE, UP 2018.C101, UniLaSalle, 19 rue Pierre Waguet, BP 30313, 60026 Beauvais, France; alicia.ayerdi-gotor@unilasalle.fr

**Keywords:** *Ulva lactuca*, fatty acids, phenolic compounds, antioxidant activity, enzyme inhibition, anti-diabetic properties, molecular docking

## Abstract

In this research, the chemical compositions of various extracts obtained from *Ulva lactuca*, a type of green seaweed collected from the Nador lagoon in the northern region of Morocco, were compared. Their antioxidant and anti-diabetic properties were also studied. Using GC–MS technology, the fatty acid content of the samples was analyzed, revealing that palmitic acid, eicosenoic acid, and linoleic acid were the most abundant unsaturated fatty acids present in all samples. The HPLC analysis indicated that sinapic acid, naringin, rutin, quercetin, cinnamic acid, salicylic acid, apigenin, flavone, and flavanone were the most prevalent phenolic compounds. The aqueous extract obtained by maceration showed high levels of polyphenols and flavonoids, with values of 379.67 ± 0.09 mg GAE/g and 212.11 ± 0.11 mg QE/g, respectively. This extract also exhibited an impressive ability to scavenge DPPH radicals, as indicated by its IC_50_ value of 0.095 ± 0.12 mg/mL. Additionally, the methanolic extract obtained using the Soxhlet method demonstrated antioxidant properties by preventing β-carotene discoloration, with an IC_50_ of 0.087 ± 0.14 mg/mL. Results from in-vitro studies showed that extracts from *U. lactuca* were able to significantly inhibit the enzymatic activity of α-amylase and α-glucosidase. Among the various extracts, methanolic extract (S) has been identified as the most potent inhibitor, exhibiting a statistically similar effect to that of acarbose. Furthermore, molecular docking models were used to evaluate the interaction between the primary phytochemicals found in these extracts and the human pancreatic α-amylase and α-glucosidase enzymes. These findings suggest that *U. lactuca* extracts contain bioactive substances that are capable of reducing enzyme activity more effectively than the commercially available drug, acarbose.

## 1. Introduction

*Ulva lactuca*, also known as sea lettuce, forms a very thin blade made up of two layers of cells, orbicular in shape and transparent, reminiscent in texture of lettuce [1]. The thallus, which is flat and entire, can measure up to 10 cm in width and is attached by a small, disc-shaped spike. The cells of the bi-stromatic blade are rectangular and contiguous, 20 to 23 µm long and 20 to 21 µm high, and contain a single parietal chloroplast. The *U. lactuca* is an alga found along coastlines worldwide [2,3,4]. This macroalga is the only one capable of causing dense blooms [5,6]. Although it can grow at depths of up to 15 m, it is typically found at a depth of 1 m due to its requirements for light color and intensity [5]. The growth rate of this alga depends on the amount of light, nitrogen, and sporulation. Additionally, the presence of epiphytes and grazers can limit its growth [7]. Due to its two-cell-layered thallus, the surface area per unit volume of *Ulva lactuca* is highly significant, enabling it to absorb large quantities of nutrients through its cell wall. This absorption capacity is particularly high when growth rates are elevated, and under favorable conditions, *U. lactuca* can absorb up to between four and six times more nutrients than other algal species [8]. In response to high nutrient levels, *U. lactuca* increases its nutrient absorption, grows rapidly, and stores intracellular nutrients for future growth [9].

Insoluble fibers, such as hemicellulose, cellulose, and lignin account for around a third of the seaweed’s dry matter, which is significantly lower than in terrestrial plants [10]. Cellulose, the most abundant organic compound on earth, is present in both marine and terrestrial plants [11]. However, the cellulose of algal species forms a more porous network, which differs considerably from the cellulose of higher plants [12]. Cellulose contents vary considerably from one algal species to another: crude cellulose contents of 11%, but also 0.85% of dry weight have been found [13]. Similarly, cellulose concentrations vary according to the different parts of the alga (frond or stipe) [14]. In comparison, the cell walls of terrestrial plants are composed of a high content of cellulose (45% DW), hemicellulose (18% DW), and lignin (20% DW), which play an essential role in vessel construction, strengthen the entire plant structure, and prevent the plant collapsing into the air [11,15,16]. Algae, on the other hand, need no support as they grow in an aquatic environment. The cellulose content of *Ulva lactuca* is significantly lower than that of terrestrial plants, and although compounds comparable to lignin have been found in primitive algae, most seaweeds contain little or no lignin [11,14,16]. The lignin content of *Ulva lactuca* is only 1.6%, which is extremely low compared with the 17–24% found in the grass and legume families [17]. *Ulva lactuca* polysaccharides are easily hydrolyzed due to their low lignin content, enabling high concentrations of bioethanol to be obtained per unit weight [14].

Seaweed is rich in nutrients such as vitamins and minerals, while being low in fat and calories compared to foods of animal origin, giving it a high nutritional value [18]. In particular, *Ulva lactuca* is a source of high-quality protein containing 17 different amino acids, including all the essential ones, making it excellent for human consumption [19]. In addition to its food potential, seaweed can also be used to produce biofuels. *Ulva lactuca* is an efficient biomass source for biofuel production, with a high potential yield of 24 tons of dry biomass per hectare per year, similar to that of sugar beet and three times higher than brown seaweed [20].

The aim of the present study was to undertake a comprehensive chromatographic analysis of the fatty acids and polyphenols present in *U. lactuca* extracts. For this purpose, two extraction techniques were employed, using solvents with differing polarities for comparative analysis. Furthermore, the research aimed to evaluate the antioxidant potential of the extracts and assess their impact on the activities of pancreatic α-amylase and α-glucosidase. The results of these analyses will provide a better understanding of the potential health advantages of *U. lactuca* extracts sourced from the Marchica lagoon, as well as their potential applications in food and pharmaceutical industries.

## 2. Results

### 2.1. Yields, Phenols, and Flavonoid Contents

This research aims to assess the efficacy of different extraction procedures by using two distinct methods, namely Soxhlet (90 °C) and maceration (25 °C). The evaluation was conducted using four solvents with increasing polarity, including hexane, ethyl acetate, methanol, and water. The phenols, flavonoids, and extraction yield of *U. lactuca* extracts were studied, and the results are presented in Table 1.

In terms of maceration extraction, the aqueous extract demonstrated the highest extraction yield at 9.45 ± 0.05%, followed by the methanolic extract at 1.18 ± 0.09%. Furthermore, the methanolic extract obtained through Soxhlet extraction had the highest polyphenolic yield with 4.21 ± 0.04%, followed by the hexanic extract with 2.23 ± 0.07%. The ethyl acetate extract had the lowest yield at 1.34 ± 0.06%.

The total phenolic content of the extracts was quantified in terms of gallic acid equivalent (GAE), as presented in Table 1. The highest total polyphenolic content was observed in the aqueous extract obtained through maceration, registering a value of 379.67 ± 0.09 mg GAE/g. Following this, the ethyl acetate extract exhibited a total phenolic content of 198.09 ± 0.11 mg GAE/g and 145.74 ± 0.15 mg GAE/g, using the maceration and Soxhlet extraction methods, respectively. These findings indicate the efficacy of both water and ethyl acetate as solvents for phenolic compound extraction from *U. lactuca*, regardless of the extraction method employed. In contrast, the methanolic extracts displayed the lowest total phenolic content, with values of 47.53 ± 0.05 mg GAE/g and 32.46 ± 0.08 mg GAE/g.

The flavonoid content of the different extracts was measured in terms of quercetin equivalent (QE), as detailed in Table 1. Flavonoid concentrations ranged from 20.24 ± 0.04 mg QE/g to 212.11 ± 0.11 mg QE/g. The highest concentration was observed in the aqueous extract obtained through the maceration method. Following this, the ethyl acetate extract exhibited concentrations of 102.1 ± 0.09 mg QE/g and 65.27 ± 0.05 mg QE/g, utilizing the maceration and Soxhlet extraction techniques, respectively.

### 2.2. Fatty Acid Analysis

The chemical compositions of *Ulva lactuca* hexane and ethyl acetate extracts are presented in Table 2. Analysis by GC–MS revealed the presence of nine saturated and unsaturated fatty acids, the content of which varied according to the solvent used. Compared to terrestrial plants, marine algae have a unique fatty acid (FA) profile. Fatty acid composition in samples is expressed in terms of total fatty acids. The percentages of saturated fatty acids (SFA), monounsaturated fatty acids (MUFA), and polyunsaturated fatty acids (PUFA) vary according to the solvent and extraction method used. The main constituents of *Ulva lactuca* extracts were palmitic acid, eicosenoic acid, and linoleic acid.

In the hexane extract, eicosenoic acid (C20:1) was the most abundant fatty acid obtained via maceration extraction, with 35.44%, followed by palmitic acid (16:0), with a high amount also at 28.05%, and by linoleic acid, with relatively the same concentration in both extraction methods at 23–25%. However, the hexane extract (46.81%) and the ethyl acetate extract (49.92%) obtained via Soxhlet exhibited the highest concentration of palmitic acid. Specifically, in the ethyl acetate extract obtained through Soxhlet, oleic acid (C18:0) was exclusively detected at a concentration of 28.60%, while linoleic acid (C18:2) showed a relatively high concentration of 23.85%. Nevertheless, margaric acid, linolenic acid, and stearic acid were found in low amounts in only one extract of *U. lactuca*, ranging from 3.42% to 6.9%. 7,10-Hexadecadienoic acid and palmitoleic acid accounted for 2.60% to 24.72% of the total fatty acids.

The hexane extract and the ethyl acetate extract obtained through the maceration method revealed the highest UFA/SFA ratio on the order of 2. These results draw attention to the possibility of using these extracts as a natural source of polyunsaturated fatty acids for nutritional, cosmetic, or pharmaceutical purposes.

### 2.3. HPLC Analysis of U. lactuca Extracts

High-performance liquid chromatography coupled with a diode array detector (HPLC-DAD) was utilized to analyze the chemical composition of the ethyl acetate and methanol extracts derived from *U. lactuca*. The obtained results were compared with standard compounds based on retention time and ultraviolet spectra; the findings are presented in Table 3. The investigation identified nine phenolic acids (4-hydroxybenzoic acid, chlorogenic acid, sinapic acid, cinnamic acid, gallic acid, caffeic acid, syringic acid, *p*-coumaric acid, and salicylic acid) along with ten flavonoids (catechin, vanillin, quercetin-3-glucoside, 7,3′,4′-flavon-3-ol, rutin, quercetin, naringin, apigenin, kaempferol, flavone, and flavanone).

The most abundant phenolic compounds found in *Ulva lactuca* extracts were sinapic acid (10.46%) in EAcE (M), (14.17%) in EAcE (S), (14.43%) in EM (M), and (12.69%) in EM (S); 7,3′,4′-flavon-3-ol (10.94%) in EAcE (S); naringin (12.81%) in EAcE (M) and (13.11%) in EM (M); and rutin (10.30%) in EAcE (S) and (12.42%) in EM (S). We also found salicylic acid (11.83%) in ME (M), quercetin (10.10%) in EAcE (M) and (9.89%) in EAcE (S), cinnamic acid (8.94%) in EAcE (M) and (9.44%) in EAcE (S), apigenin (11.58%) in EAcE (S), flavone (11.29%) in EAcE (S), and flavanone with a value of (13.62%) in EAcE (M) and (22.72%) in EAcE (S).

In this study, the results revealed that naringin predominated in EAcE (M), while sinapic acid was the majority compound in EAcE (S), EM (M), and EM (S). In contrast, flavanone was found to be the main compound in ME (M) and ME (S). Other compounds were identified in *Ulva lactuca* extracts but at low percentages, namely gallic acid, catechin, 4-hydroxy-benzoic acid, chlorogenic acid, caffeic acid, syringic acid, vanillin, *p*-Coumaric acid, quercetin 3-glucoside, and kaempferol.

### 2.4. Antioxidant Activity

Antioxidants have garnered considerable attention in research owing to their capacity to maintain food quality and their potential in treating diseases linked to oxidative stress. In this study, we employed the DPPH free radical scavenging assay and the β-carotene bleaching assay. The IC_50_ values for *U. lactuca* ethyl acetate extract (EAcE), methanol extract (ME), and aqueous extract (AQE) are detailed in Table 4.

The results indicate that the highest antioxidant activity was observed in the case of the aqueous extract, with a value of 0.09 mg/mL, compared to the reference antioxidant, ascorbic acid, which exhibited an IC_50_ value of 0.06 mg/mL. The findings also suggest that ethyl acetate extracts (0.55–0.62 mg/mL) generally exhibit antioxidant activity similar to methanolic extracts (0.59–0.65 mg/mL), regardless of the extraction method employed. This may be explained by the fact that antioxidant compounds present in water are more effectively solubilized and extracted than those in organic solvents.

The aqueous extract obtained by maceration showed high antioxidant activity by the DPPH method, compared with ascorbic acid used as a reference. The richness of this extract in polyphenols and flavonoids could explain this high activity [21].

### 2.5. In-Vitro α-Amylase Inhibition

Table 5 presents the effect of *Ulva lactuca* extracts on α-amylase and α-glucosidase inhibition activity, using acarbose as a positive control. In-vitro studies were conducted to examine the impact of various doses of the extracts on α-amylase enzymatic activity. Results showed that all extracts significantly inhibited α-amylase enzymatic activity at all tested doses. Among the different samples, the concentration of 1.14 mg/mL had the most active effect, exhibiting inhibitory activities of 57.64 ± 0.47 for EACE (M), 57.61 ± 0.21 for EACE (S), 57.42 ± 0.10 for ME (M), 67.06 ± 0.06 for ME (S), and 57.67 ± 0.34 for AQE (M). The results revealed that EACE (M), EACE (S), ME (M), and AQE (M) possess the same inhibitory effect among them, with lower inhibitory activity than acarbose (*p* < 0.001 for EACE (S), ME (M), and AQE (M); and *p* < 0.01 for EACE (M) compared with acarbose). However, ME (S) exhibits a higher inhibitory effect compared to the other extracts and has an effect statistically similar to acarbose.

As for the effect of *Ulva lactuca* extracts on pancreatic α-glucosidase inhibitory activity, using acarbose as a positive control, the results showed that EM (S) was the most effective, closely followed by EAcE (S), EAcE (M), EM (M), and finally AQE (M).

### 2.6. Molecular Modeling Studies

The study of molecular docking provides valuable insights into the regulation of enzymes involved in carbohydrate digestion, such as α-amylase and α-glucosidase [22]. These enzymes play crucial roles in breaking down carbohydrates during digestion. Specifically, α-amylase acts on starch, breaking it down into smaller carbohydrates, while α-glucosidase further breaks these down into simple sugars like glucose [23]. The absorption of these sugars into the bloodstream can contribute to elevated blood sugar levels in individuals with diabetes if not properly regulated [24]. To address this issue and manage blood sugar levels effectively, some individuals with diabetes may consider the use of α-amylase or α-glucosidase inhibitors, which work to slow down the digestion and absorption of carbohydrates [25]. Molecular docking studies can enhance our understanding of the interactions between these inhibitors and the targeted enzymes, providing valuable insights for potential therapeutic interventions. All molecules identified in *Ulva lactuca* extracts by HPLC-DAD exhibited remarkable inhibitory activity against α-amylase and α-glucosidase compared to acarbose (standard). It was observed that all these compounds remained stable in the active sites, presenting particularly high energy values, except for cinnamic acid in both proteins. The presence of negative and low docking score values suggests that the compounds engaged in robust and advantageous binding interactions (Table 6).

**Figure 1:**
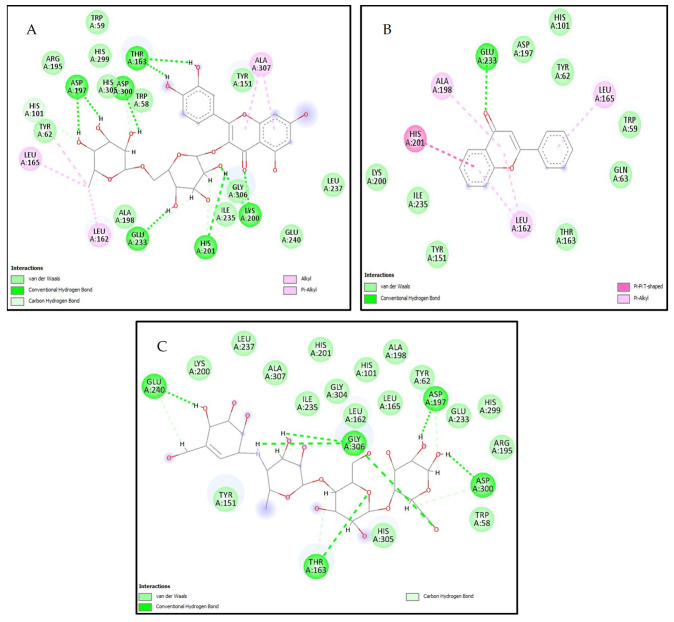
2D intermolecular interactions between (**A**) rutin, (**B**) flavone, and (**C**) acarbose (standard) with the active site of α-Amylase (PDB: 1B2Y) protein.

**Figure 2:**
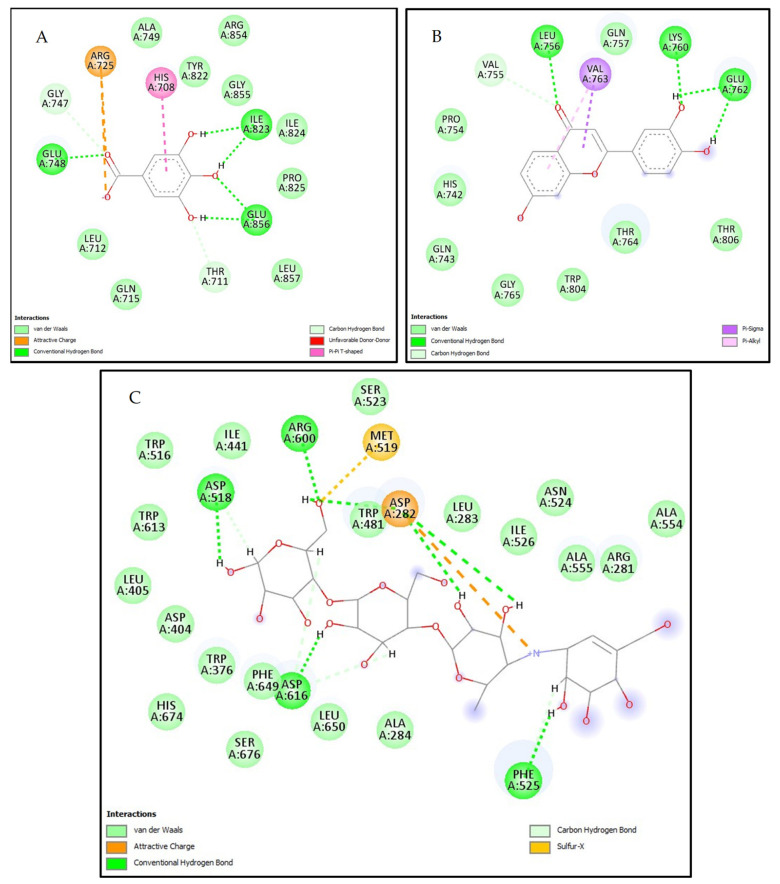
2D intermolecular interactions between (**A**) gallic acid, (**B**) 7,3′,4′-flavon-3-ol, and (**C**) acarbose (standard) with the active site of α-glucosidase (PDB: 5NN8) protein.

The compounds exhibiting the highest stability in the active site of α-amylase are rutin and flavone, with docking scores of −6.807 kcal/mol and −5.994 kcal/mol, respectively. These values surpass the docking score of acarbose, which is −4.877 kcal/mol. Notably, these compounds form various interactions through the active site, such as conventional hydrogen bonds, carbon–hydrogen bonds, pi–alkyl, alkyl, pi–pi T-shaped, and van der Waals interactions with the amino acid residues ALA307, THR163, ASP300, ASP197, TYR62, LEU165, GLU165, LEU162, GLU233, LYS200, GLU233, ALA198, HIS201, GLY306, and GLU240 (Figure 1). On the other hand, the compounds displaying the highest stability in the active site of α-glucosidase are gallic acid and 7,3′,4′-trihydroxyflavone, with docking scores of −7.334 kcal/mol and −6.620 kcal/mol, respectively. These values surpass the docking score of acarbose, which is −4.925 kcal/mol. Notably, these compounds form various interactions within the active site, such as conventional hydrogen bonds, carbon–hydrogen bonds, pi–alkyl, pi–sigma interactions, attractive charge interactions, sulfur–x bonds, and van der Waals forces with amino acid residues following ILE823, GLU856, HIS708, ARG725, GLU748, GLY747, THR711, VAL756, VAL763, LYS760, GLU762, ASP518, ARG600, MET519, ASP282, ASP616, and PHE525 (Figure 2). While in-silico studies have shown promising results regarding the inhibitory potential of the identified compounds in *Ulva lactuca* on α-amylase and α-glucosidase proteins, further research is needed to determine their therapeutic effectiveness in a clinical setting. Clinical trials involving human subjects are essential to assess the safety and pharmacokinetics of these compounds [26].

## 3. Discussion

In this study, the results show that using polar solvents like water and methanol produced greater extraction yields compared to less polar solvents like ethyl acetate and hexane. This suggests that increasing the polarity of the solvent leads to an increase in the extraction yield, which is confirmed by previous research [27,28]. In addition, polar solvents, like water, can extract phenolic compounds that are attached to sugars or proteins, saponins, glycosides, organic acids, and selenium [29].

The phenolic content values found in *Ulva lactuca* are higher than those obtained by Oucif et al. [30] (4.27–8.69 mg GAE/g DE) as is the flavonoid content (4.66–1.0 mg GAE/g DE) in the methanolic and aqueous extract. The observed difference can be explained by several factors, such as the origin of the algae sample, the extraction method used, the stage of maturity of the algae, the growth conditions, or the type of solvent used for extraction [31,32].

The analysis of the chemical composition of EAcE and HE by gas chromatography–mass spectrometry (GC–MS) highlights the predominance of palmitic acid, eicosenoic acid, and linoleic acid as major compounds. Palmitic acid exhibits inhibitory effects on prostate cancer cell proliferation and metastasis by suppressing the PI3K/Akt pathway [33]. Omega-3 fatty acids, particularly linolenic acid, play a vital role in regulating blood glucose levels by enhancing insulin secretion from pancreatic beta cells in the islets of Langerhans [34]. Linoleic acid, an essential fatty acid, demonstrates hypocholesterolemic effects [35] and facilitates glucose uptake by C2C12 muscle cells [36]. Oleic acid has been observed to stimulate insulin secretion in glucose-sensitive INS-1 cell lines, notably even in the presence of the inflammatory cytokine TNF-α [37]. Stearic acid promotes glucose uptake into adipocytes by activating insulin/insulin receptor signaling while inhibiting PTP1B [38]. The expression of GLUT4 transporter and glucose uptake facilitation in 3T3-L1 adipocytes are significantly influenced by polyunsaturated fatty acids, leading to increased abundance of both GLUT4 and GLUT1 transporters [39,40]. Furthermore, the impact of eicosenoic acid, specifically 7,10-hexadecadienoic acid, on diabetes remains relatively understudied compared to other omega fatty acids such as omega-3 and omega-6 fatty acids.

It is imperative to acknowledge that the composition and ratio of fatty acids in a diet can profoundly influence its overall impact on health [41,42]. For instance, elevated levels of saturated fatty acids (SFAs) have been correlated with an increased risk of cardiovascular disease, while polyunsaturated fatty acids (PUFAs) have been associated with a reduced risk. Conversely, monounsaturated fatty acids (MUFAs) are generally regarded as beneficial for health due to their potential to lower cholesterol levels and mitigate the risk of heart disease [43]. PUFAs, constituting a significant portion of cell membrane phospholipids, play a vital role in human metabolism [44,45]. Their metabolic significance is underscored by their diverse biological properties, encompassing antibacterial activity [46,47,48], anti-inflammatory effects [49,50], antioxidant capacity [51], potential for preventing cardiovascular disease [52], and inhibition of tumor growth [53,54].

Other studies found significant variations in the FA composition of the same species of green seaweed, collected from different locations. This poses a challenge in establishing a direct correlation between a specific FA profile and a particular green seaweed species. For example, *U. lactuca* collected from the North California coast in November exhibited 11% α-linolenic acid, 22% stearidonic acid (18:4 ω3), 1% oleic acid (18:1 ω9), and 24% palmitic acid [55]. On the other hand, *U. lactuca* collected from the North Sea in September/October had 20% α-linolenic acid, 8% stearidonic acid, 20% oleic acid, and 12% palmitic acid [56].

The major phenolic molecules of the different EAcE and ME were determined through HPLC analysis. The study revealed that *U. lactuca* extracts are an excellent source of flavonoids and polyphenols. Flavonoids and their glycosides represent a significant class of plant secondary metabolites. These compounds are widely distributed in nature and are of particular interest due to their antioxidant properties and their potential role in preventing various diseases, including cancer, cardiovascular diseases, neurodegenerative diseases, diabetes, and osteoporosis [57]. Flavonols, also referred to as hydroxyflavones, are distinguished from flavones by the presence of a hydroxy group at position 3 in the chromen-4-one ring [58]. Despite their structural similarity, natural flavonols do not originate from chalcones through flavones as intermediates but rather through an alternative biochemical pathway involving different enzymes, via flavanones. Flavanones serve as common precursors for both flavones and flavonols [59].

Rutin (30,40,5,7-tetrahydroxy-flavone-3-rutinoside) is a flavonol glycoside with documented clinically relevant properties, potentially advantageous in disease prevention and genome stability preservation [60]. The Dietary Supplement Label Database currently lists over 860 products containing rutin marketed in the U.S. [61]. Rutin supplementation is particularly recommended for managing various conditions such as varicose veins, internal bleeding, or hemorrhoids. Common oral dosages range from 500 mg to 2000 mg daily and can be safely continued for extended periods, up to 6 months [62].

Salicylic acid (SA) is a widely used nonsteroidal anti-inflammatory drug (NSAID) known for its antibacterial, anti-biofilm-formation, antipyretic, and anticoagulation properties [63]. Apigenin and nobiletin have been shown to modulate the expression of crucial inflammatory signaling pathways, including nuclear factor erythroid 2-related factor 2 (Nrf2) and nuclear factor kappa-light-chain-enhancer of activated B cells (NF-κB). The antioxidant attributes of various natural flavones stem from their capacity to regulate the expression of Nrf2/heme oxygenase-1 (HO-1), thereby reducing levels of free radicals and oxidative stress.

Sinapic acid stands out as one of the four most prevalent hydroxycinnamic acids, distributed widely across the plant kingdom. Numerous researchers have suggested its potency as an antioxidant [64,65,66,67]. Its efficacy is purported to surpass that of ferulic acid [68], another hydroxycinnamic acid extensively utilized as a natural antioxidant in various food, beverage, and cosmetic products [69], and is comparable to caffeic acid [64,70,71]. Sinapic acid exhibits antimicrobial [72,73,74,75,76,77], anti-inflammatory [78], anticancer [79], and anti-anxiety properties [80].

The DPPH assay relies on the capacity of the stable free radical DPPH (•) to reduce its color intensity in the presence of antioxidants. This radical possesses an unpaired electron, which imparts a deep purple coloration that is attenuated in the presence of antioxidants [81]. The DPPH assay is commonly utilized as a tool to evaluate the antioxidant activity of various plant extracts [82,83].

The ability of *U. lactuca* extracts to arrest or retard lipid peroxidation was assessed using the β-carotene molecule bleaching method. In this test, linoleic acid oxidation generates peroxide radicals following the abstraction of hydrogen atoms from linoleic acid methylene groups. These free radicals then oxidize the highly unsaturated β-carotene, causing it to lose its red color, which is monitored spectrophotometrically at 470 nm. However, the presence of an antioxidant could neutralize linoleic acid-derived free radicals and thus prevent β-carotene oxidation and bleaching.

The findings indicated that extracts derived from the green algae *U. lactuca*, exhibiting high total phenol content, displayed notable antioxidant activity. This suggests that polyphenols found in algae may play a pivotal role in the radical scavenging properties of these extracts. Existing literature has highlighted a strong correlation between antioxidant activity and the presence of polyphenols and flavonoids. Consequently, these algae represent a valuable reservoir of bioactive compounds and hold considerable potential for antioxidant activity, as demonstrated by the enhanced efficacy observed with heating during Soxhlet extraction.

It is important also to note that antioxidant activity determined by various methods may provide inconsistent findings [84]. The differences in the findings of the two experiments may be traced to the distinct processes involved.

The α-amylases (1,4-α-D-glucosyl-hydrolase) are enzymes present in plants, animals, and microorganisms [85]. They constitute a part of both salivary and pancreatic secretions, playing a crucial role in the breakdown of complex carbohydrates [86]. Pancreatic α-amylase, functioning as a digestive enzyme, initiates the hydrolysis of starch, converting it into maltose and eventually glucose. This process involves the hydrolysis of the α-1,4-glucan bonds present in starch and glycogen, resulting in the production of glucose and maltose [87]. α-glucosidases, positioned at the brush border of enterocytes, serve as digestive enzymes catalyzing the hydrolysis of α-(1,4) glucosidic bonds in undigested dietary disaccharides reaching the jejunum without modification, as well as the residues generated from starch digestion. This leads to the formation of absorbable glucose within intestinal cells [88]. Pancreatic α-amylases and intestinal α-glucosidase inhibition activity become part of a strategic approach to delay the glucose absorption from the digestive tract, which helps prevent rapid spikes in blood sugar levels after meals, contributing to better overall glycemic control [89]. Medications such as acarbose, known as α-amylases and α-glucosidase inhibitors, are commonly employed to achieve this goal [90]. Nevertheless, despite the positive impact of this treatment on glycemic control and overall body balance, it is accompanied by side effects, including diarrhea and flatulence [91]. This prompts individuals to actively seek natural alternatives. Among the natural sources that have shown an effect on diabetes, marine algae are noteworthy [92]. In our research, we explored the impact of different extracts from marine algae *Ulva lactuca* to assess their inhibitory and therapeutic potential against these two enzymes.

The main findings of the in-vitro study on the inhibitory effect of α-amylase and α-glucosidase summarize that each *Ulva lactuca* extract showed significant inhibition of the enzymatic activity of α-amylase at all tested doses. The IC_50_ values revealed similar inhibitory effects among EACE (M), EACE (S), ME (M), and AQE (M), all exhibiting lower activity compared to acarbose, the reference molecule. However, ME (S) demonstrated a higher inhibitory activity than the other extracts, displaying a statistically similar effect to acarbose, which means that the extraction method of this alga does not influence enzyme inhibition, except for the methanolic extract. It is observed that extraction using Soxhlet yields better results than maceration. This can be explained by the fact that the Soxhlet method extracted a much higher amount of active principles responsible for the beneficial effect compared to maceration. Our results are in agreement with [93], which suggests that the Soxhlet methanolic extract of Galium aparine L. has demonstrated a more pronounced α-amylase inhibition effect and radical scavenging activity than other extracts (maceration and ultrasonic). This effect is attributed to the high concentration of phenolic compounds, flavonoids, and tannins. In fact, our results align with a previous study, which reported that the IC_50_ values for α-amylase inhibition by aqueous extracts of seaweed *Ulva lactuca*, Sargassum polycystum, G. Gracilaria edulis, and Gracilaria corticate are, respectively, 67 µg/mL, 60 µg/mL, 83 µg/mL, and 82 µg/m [94]. Furthermore, the study added that the extraction using ethyl acetate of the green seaweed *Ulva fasciata* exhibited lower inhibitory activity against α-amylase (IC_50_ = 69.12 g/mL) compared to the positive control, acarbose (IC_50_ = 49.34 µg/mL) [95].

*Ulva lactuca* is rich in minerals, vitamins, and polysaccharides that exhibit several bioactive properties, including anticoagulant, antiviral, anti-inflammatory, immunomodulatory, and antioxidant activities [96]. Phenolic compounds, notably tannins and flavonoids such as quercetin, kaempferol, and apigenin, play a crucial role as antioxidant and anti-diabetic agents, modulating oxidative stress and inflammation [97]. According to Sok Yen et al. [98], flavonoids exhibit both hypoglycemic and antioxidant effects in diabetic animals. Moreover, they have been found to inhibit both α-amylase and α-glucosidase activities with IC_50_ values of 770 μg/mL, 32 μg/mL for quercetin and 287.53, 231.13 μg/mL for apigenin, respectively [90,99]. Fatty acids, including linoleic acid, gamma-linolenic acid, oleic acid, linolelaidic acid, and α-linoleic acid, are also reported as a main component of *Ulva lactuca*. Oleic acid demonstrates the most potent anti-α-glucosidase activity, followed by linoleic acid. Their activities proved more potent than acarbose [100]. In addition, *Ulva lactuca* contains pigments such as chlorophylls that play a role in photosynthesis and exhibit antioxidant properties. Also, it contains carotenoids such as beta-carotene, lutein, and zeaxanthin that have hypoglycemic activity and a strong antioxidant effect. It may also play an essential role in diabetic complications [101]. All these biomolecules are collectively contributing to the nutritional and therapeutic potential of *Ulva lactuca*.

## 4. Materials and Methods

### 4.1. Chemicals and Reagents

Analytical-grade chemicals and reagents were procured from Merck (Darmstadt, Germany) and Carl Roth GmbH (Karlsruhe, Germany) to determine the total phenolic and flavonoid components. Methanol, ethyl acetate, and N-hexane were acquired from Merck (Darmstadt, Germany). α-amylase, α-glucosidase, β-carotene, 1,1-Diphenyl-2-picrylhydrazyl (DPPH•), acarbose, and 3,5-Dinitrosalicylic acid (DNSA) were obtained from Merck (Sigma–Aldrich, St. Louis, MO, USA). Phenolic standards including ascorbic acid, kojic acid, gallic acid, apigenin, succinic acid, cholesterol, and tannic acid were sourced from Merck and Carl Roth GmbH (Karlsruhe, Germany).

### 4.2. Plant Material and Extraction

In April 2021, the green algae species *U. lactuca* was harvested from the Nador lagoon located at 35°08′26.9″ N 2°29′09.6″ W in northern Morocco. The classification of this algae species was carried out by Dr. M. Ramdani, who is affiliated with the Faculty of Sciences at the University Mohammed I in Oujda, Morocco.

**Figure 3:**
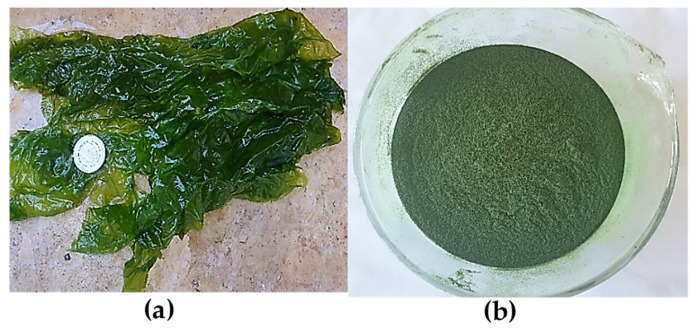
(**a**) *U. lactuca* before drying, (**b**) powder of dried *U. lactuca*.

The collected seaweed sample was transported to the laboratory for further processing. *U. lactuca* was carefully cleaned and rinsed with distilled water before being exposed to light for 48 h. The dried sample was then placed in an oven at 35 °C for 24 h. The seaweed was then lyophilized and ground into a fine powder for extraction purposes, as illustrated in Figure 3. To extract the valuable components, we utilized maceration and Soxhlet techniques with four solvents: hexane, ethyl acetate, methanol, and distilled water. The resulting extracts were then filtered using a glass filter crucible (50 mL, with porosity 4, Isolab, Wertheim, Germany) and placed in flasks. The extracts were then dehydrated using a rotary evaporator (Laborota 4000, Heidolph Instruments, Schwabach, Germany). This thorough extraction process ensures that the resulting extracts are of the highest quality, making them ideal for further studies, and the extraction yield was calculated using the following equation:

$$\mathrm{Yield}\;\left(\%\right)=\frac{mass\;dried\;extract\;\left(g\right)}{mass\;dried\;matrix\;\left(g\right)}\times 100$$

The recorded datum is the median of three extraction replicates.

#### 4.2.1. Maceration Extraction

**Figure 4:**
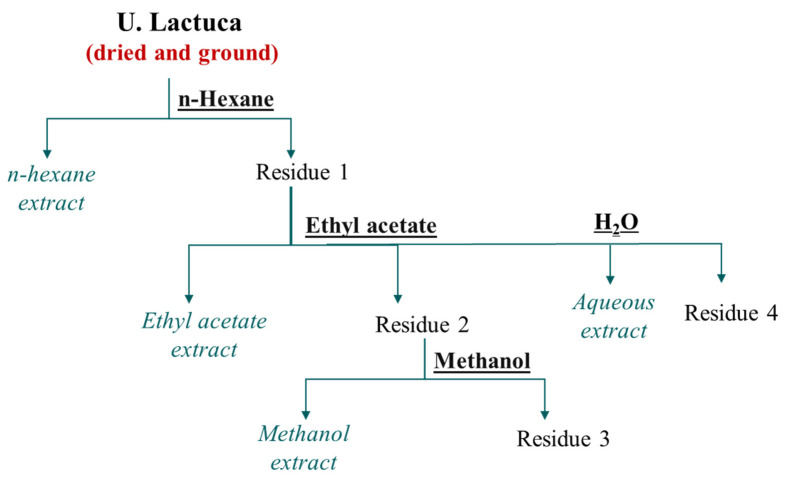
Extraction process of *U. lactuca* with various solvents.

To extract soluble compounds from a solid substance, maceration is the most common method, involving immersion in a cold liquid. In this case, extracts were prepared successively by stirring 100 g of macroalgae powder with 200 mL of solvent of increasing polarity (99% n-hexane for 2 h, ethyl acetate for 24 h, methanol for 24 h, and distilled water for 24 h) at room temperature. This process is illustrated in Figure 4.

#### 4.2.2. Soxhlet Extraction

In order to extract active compounds from marine macroalgae, we utilized the alternative technique, namely the Soxhlet extraction method. This method involves employing a Soxhlet chamber, an extraction flask, and a condenser [102]. The process begins by placing 35 g of marine macroalgae powder into an extraction thimble, which is then combined with 300 mL of a selected solvent (such as hexane, ethyl acetate, or methanol) in the extraction flask. The duration of the Soxhlet extraction process is determined by the time it takes to extract all the soluble compounds with affinity for the solvent at a given temperature.

### 4.3. Phytochemicals Compounds

#### 4.3.1. Quantification of Total Phenolic Constituents

The study aimed to determine the total amount of polyphenols present in *U. lactuca* extracts. To achieve this, a modified Folin–Ciocalteu method was used. In this method, 200 µL of the extract solution with a concentration of 1 mg/mL was mixed with 1000 µL of Folin–Ciocalteu reagent dissolved in distilled water, followed by the addition of 800 µL of sodium carbonate (75 g/L). The resulting mixture was then incubated at room temperature for one hour in the dark. After incubation, absorbance readings were taken at 765 nm using a spectrophotometer, with ethanol serving as a control. To generate calibration curves, the concentration of gallic acid was altered (ranging from 0 to 0.1 mg/mL). All experiments were conducted in triplicate to obtain mean values and their corresponding standard deviations. The total amount of phenolic compounds was expressed in milligrams of gallic acid equivalents per gram of dry extract (mg GAE/g) [103].

#### 4.3.2. Measurement of Total Flavonoid Contents

We combined 200 µL of each *U. lactuca* extract, 1000 µL of distilled water, and 50 µL of NaNO_2_ (5%). Subsequently, after a 6 min incubation period, 120 µL of AlCl_3_ (10%) was introduced, followed by a further 5 min incubation period at room temperature in darkness. This was followed by the addition of 400 µL of 1 M NaOH and 230 µL of distilled water. The absorbance was then measured at 510 nm. To construct the calibration curve, various concentrations of quercetin solution (ranging from 0 to 0.1 mg/mL) were utilized as standards. Each measurement was conducted in triplicate to ensure result reproducibility.

### 4.4. Fatty Acid GC–MS Analysis of U. lactuca Extracts

In line with the methodology outlined in Loukili et al. [42], methyl esters of hexane fatty acids and ethyl acetate were extracted from *U. lactuca* with certain adaptations. The Shimadzu GC system (Kyoto, Japan) equipped with a BPX25 capillary column featuring a 5% diphenyl and 95% dimethylpolysiloxane phase (30 m × 0.25 mm × 0.25 µm) (Kyoto, Japan) coupled with a QP2010 MS was utilized for the characterization and identification of fatty acids. Helium gas (99.99%) served as the mobile phase with a flow rate set at 3 L/min. The temperature settings for the injection, ion source, and interface were maintained at 250 °C. The column oven temperature program began at 50 °C for 1 min, followed by a gradual increase to 250 °C at a rate of 10 °C/min, and held for an additional minute. Sample components underwent ionization in electron ionization (EI) mode at 70 eV, scanning a mass range of 40 to 300 m/z. Each extract, in a volume of 1 µL, was injected in split mode, and triplicate analyses were conducted for each sample. Compound characterization relied on comparisons of retention times, mass spectra fragmentation patterns, and databases, including the National Institute of Standards and Technology’s database (NIST). Data processing was conducted using LabSolutions (version 2.5, Shimadzu, Kyoto, Japan).

### 4.5. HPLC Analyses of U. lactuca Extracts

Identification of phenolic compounds within the ethyl acetate and methanolic extracts was accomplished utilizing high-performance liquid chromatography (HPLC) employing an Agilent 1100 system (Agilent Technologies, Palo Alto, CA, USA) coupled with a diode array UV detector (Bruker, Berlin, Germany). Each extract (10 µL) underwent separation through a Zorbax XDB-C18 column (5 µm, 250 × 4.6 mm) connected to the Agilent 1100 system, preceded by a 4 × 3 mm C18 cartridge precolumn (Agilent Technologies). The elution gradient protocol employed was as follows: 0 to 5 min with 95% solvent A and 5% solvent B, 25 to 30 min with 65% solvent A and 35% solvent B, 35 to 40 min with 30% solvent A and 70% solvent B, and finally 40 to 45 min with 95% solvent A and 5% solvent B. Solvent A comprised water/methanol (9/1) with 0.1% phosphoric acid, while solvent B consisted of methanol with 0.1% phosphoric acid. The elution was carried out at a constant flow rate of 1 mL/min under a temperature of 40 °C. Spectrophotometric data were collected at wavelengths of 254 nm, 280 nm, 320 nm, 370 nm, and 510 nm. Compound identification was conducted by comparing their retention times and UV spectra with those of standard compounds.

### 4.6. Antioxidant Activity

#### 4.6.1. Scavenging 2,2-Diphenyl-1-Picrylhydrazyl Radical Test

The antioxidant activity of different extracts of *U. lactuca* was assessed using the 1,1-diphenyl-2-picrylhydrazyl (DPPH) radical bleaching method, following the protocol outlined by Brand-Williams et al. [104] with minor adjustments. The initial concentration of both the extracts and ascorbic acid was maintained at 1 mg/mL. A solution comprising 0.8 mL of samples or standard (ascorbic acid) at varying concentrations (ranging from 0.02 to 0.32 mg/mL) and 2 mL of DPPH• solution (prepared by dissolving 2 mg of DPPH• in 200 mL of MeOH) was prepared and manually agitated. Following a 30 min incubation period in darkness at room temperature, the absorbance of the samples was measured using a UV–visible spectrophotometer at a wavelength of 517 nm, relative to the blank. Each analysis was conducted in triplicate.

The inhibitory activity of the DPPH radical by a sample was determined using the following formula:

$$\mathrm{Inhibition}\;\mathrm{Percent}=\left[\frac{\left(\mathrm{Ab}-\mathrm{As}\right)}{\mathrm{Ab}}\right]\times 100$$

where Ab is absorbance of the blank and As is absorbance of a sample (or positive control).

The graph plotting inhibition percentage against extract concentration was used to calculate the IC_50_.

#### 4.6.2. β-Carotene Bleaching Assay

The antioxidant potential of *U. lactuca* extracts was assessed using the method described by [105]. This evaluation relied on the extracts’ capacity to mitigate oxidative damage to β-carotene in an emulsion, employing the carotene bleaching assay. To prepare the emulsion, 2 mg of β-carotene was dissolved in 10 mL of chloroform, supplemented with 20 mg of linoleic acid and 200 mg of Tween 80 as an emulsifier. The chloroform was evaporated under vacuum at 40 °C, followed by the addition of 100 mL of distilled water while vigorously stirring the solution. Subsequently, 0.2 mL of the emulsion was dispensed into individual test tubes, along with either the extract or a reference antioxidant solution (BHA) at a concentration of 1 mg/mL. The absorbance was recorded at 470 nm using a 96-well microplate reader at t0 (immediately after emulsion addition) and after a 2 h incubation period. All measurements were conducted in triplicate.”

The inhibition of the linoleate/β-carotene radical was determined using the following equation:

$$\mathrm{Bleaching}\;\mathrm{inhibition}\;\left(\%\right)=100-\frac{initial\;\left(\beta -\mathrm{carotene}\right)\left(t0\right)-\left(\beta -\mathrm{carotene}\right)\;after\;2\;h\;}{initial\;\left(\beta -\mathrm{carotene}\right)\left(t0\right)}\times 100$$

### 4.7. In-Vitro α-Amylase Inhibition

The inhibitory activity of various extracts of *U. lactuca* against α-amylase was assessed following the protocol outlined by Daoudi et al. [106]. A reaction mixture was prepared, comprising 0.2 mL of the sample or acarbose (utilized as a positive control at concentrations ranging from 2.27 to 0.14 mg/mL), 0.2 mL of phosphate buffer (pH 6.9), and 0.2 mL of enzyme solution (13 IU). This mixture was pre-incubated at 37 °C for 10 min before the addition of 0.2 mL of enzyme-substrate solution (1% starch dissolved in phosphate buffer). The reaction proceeded at 37 °C for 30 min. The enzymatic reaction was halted by adding 0.6 mL of DNSA reagent, followed by an incubation step at 100 °C for 8 min, followed by cooling in a cold-water bath. Absorbance was measured at 540 nm.

The inhibition percentage was calculated using the following formula:

$$\mathrm{Inhibition}\;\mathrm{activity}\;\left(\%\right)=\frac{\left(\mathrm{ODcontrol}\;540\;\mathrm{nm}-\mathrm{ODcontrol}\;\mathrm{blank}\;540\;\mathrm{nm}\right)-\left(\mathrm{OD}\;\mathrm{sample}\;540\;\mathrm{nm}-\mathrm{OD}\;\mathrm{sample}\;\mathrm{blank}540\;\mathrm{nm}\right)\;}{\mathrm{OD}\;\mathrm{control}\;540\;\mathrm{nm}-\mathrm{OD}\;\mathrm{control}\;\mathrm{blank}\;540\;\mathrm{nm}}\times 10$$

The IC_50_ of the various tests was done graphically using the function:

$$\mathrm{Inhibition}\;\mathrm{percentage}\;\left(\%\right)=f(\log\left(\mathrm{sample}\;\mathrm{concentration}\right))$$

### 4.8. In-Vitro α-Glucosidase Inhibition Assay

The effect of *U. lactuca* extracts on intestinal α-glucosidase activity was evaluated using a modified version of the protocol outlined by Hbika et al. [107]. A mixture was prepared by combining 100 mL of sucrose (50 mM), 1000 mL of phosphate buffer (50 mM, pH 7.5), and 100 mL of intestinal α-glucosidase enzyme solution (10 IU). This mixture was then supplemented with 10 mL of distilled water (as control), acarbose (as the positive control), or *U. lactuca* extract solution at a concentration of 22 mg/mL. Subsequently, the mixture was incubated for 25 min at 37 °C in a water bath. To terminate the enzymatic reaction and quantify the release of glucose, the mixture was heated at 100 °C for 5 min:

$$\mathrm{Inhibition}\;\mathrm{activity}\;\left(\%\right)=\frac{\left(\mathrm{ODcontrol}\;540\;\mathrm{nm}-\mathrm{ODcontrol}\;\mathrm{blank}\;540\;\mathrm{nm}\right)-\left(\mathrm{OD}\;\mathrm{sample}\;540\;\mathrm{nm}-\mathrm{OD}\;\mathrm{sample}\;\mathrm{blank}540\;\mathrm{nm}\right)\;}{\mathrm{OD}\;\mathrm{control}\;540\;\mathrm{nm}-\mathrm{OD}\;\mathrm{control}\;\mathrm{blank}\;540\;\mathrm{nm}}\times 10$$

### 4.9. Theoretical Study

#### 4.9.1. Ligand Preparation

To optimize and minimize the energy of the ligands (compounds) identified in *Ulva lactuca* extracts by HPLC-DAD, data were retrieved from the PubChem database (https://pubchem.ncbi.nlm.nih.gov, accessed on 21 April 2024). The LigPrep module within Maestro 12.8 (Schrodinger 2021-2) was utilized for ligand preparation. In this study, acarbose inhibitor served as the standard [108], and the energy minimization and optimization procedures adhered to the OPLS_2005 force field. Hydrogen atoms were included, and adjustments were implemented to eliminate salt and ionization effects at a pH of 7 ± 2 [109].

#### 4.9.2. Molecular Docking and Preparation of Proteins

**Figure 5:**
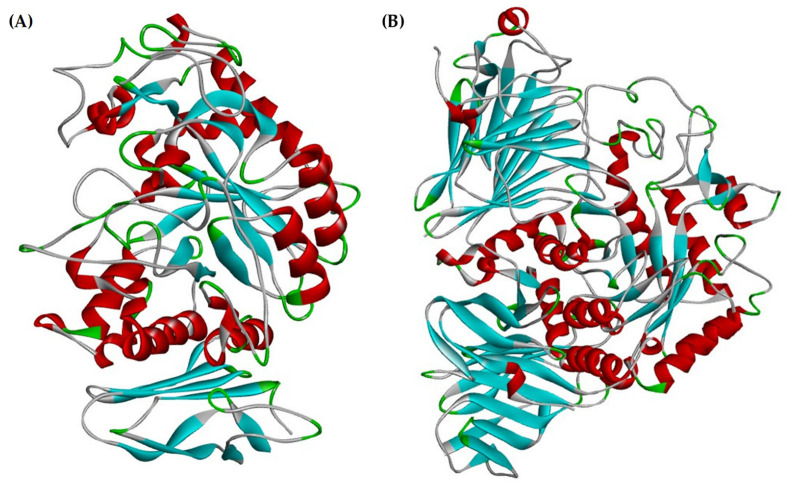
The 3D crystal structure of the proteins (**A**) α-amylase (PDB: 1B2Y) and (**B**) α-glucosidase (PDB: 5NN8).

Molecular docking was conducted using X-ray crystal structures obtained from the Protein Data Bank (PDB) [103]. Specifically, the structures utilized were “Structure of Human Pancreatic Alpha-Amylase in Complex with the Carbohydrate Inhibitor Acarbose” (PDBID: 1B2Y) with an X-ray diffraction resolution of 3.20 Å and “Crystal Structure of Human Lysosomal Acid-Alpha-Glucosidase, GAA, in Complex with Acarbose” (PDBID: 5NN8) with an X-ray diffraction resolution of 2.45 Å (Figure 5). The preparation of these protein structures involved the utilization of the Protein Preparation Wizard (Schrodinger 2021-2) [110]. During this process, ligand and water atoms were removed, and non-polar hydrogens were consolidated. The active site was defined as the target center. To establish an optimal docking environment, the central grid box dimensions were configured to encompass all atoms of the ligand set, with 20 points allocated for each axis (x, y, and z). Energy minimization was performed with default settings, constraining the root-mean-square deviation (RMSD) to 0.3 Å. The standard precision (SP) glide score was employed for predicting binding and selecting anchored poses. The output docking scores were expressed as affinity binding in kcal/mol. Subsequently, the protein structure underwent further minimization using the OPLS_2005 force field. Biovia Discovery Studio 2021 [111] was utilized for visualizing the protein–ligand complexes.

## 5. Conclusions

Seaweed has many qualities as an organic product, being rich in vitamins and minerals yet very low in calories. This study compares the chemical composition and bioactive properties of extracts from *Ulva lactuca*, a green seaweed collected from the Nador lagoon in Morocco. Using GC–MS and HPLC analyses, the fatty acid and phenolic compound content of the extracts were identified. The aqueous extract showed high levels of polyphenols and flavonoids and exhibited strong antioxidant activity against DPPH radicals. The methanolic extract demonstrated antioxidant properties by preventing β-carotene discoloration. In-vitro studies revealed that the extracts inhibited the enzymatic activity of α-amylase and α-glucosidase. Molecular docking models suggested that the extracts’ phytochemicals interacted with these enzymes more effectively than acarbose, a commercial drug. These findings highlight the potential of *U. lactuca* extracts as natural sources of bioactive compounds with antioxidant and anti-diabetic properties. Seaweed is widely perceived as natural and beneficial to health by consumers. The rise of seaweed in cosmetics products is undeniable, and its growing presence in food products is evidence of a new niche for the agri-food and pharmaceutical industries. The nutritional and therapeutic properties of algae promise to propel them towards greater valorization in the years to come, extending their influence to other sectors. This trend suggests a promising potential for future applications of algae in various economic fields.

## Figures and Tables

**Table 1:** Phenols and flavonoid contents of different extracts of *U. lactuca*.

Solvent	Extraction Methods	Polyphenols (mg GAE/g)	Flavonoids (mg QE/g)
Ethyl acetate	M	198.09 ± 0.11	102.10 ± 0.09
	S	145.74 ± 0.15	65.27 ± 0.05
Methanol	M	47.53 ± 0.05	27.18 ± 0.09
	S	32.46 ± 0.08	20.24 ± 0.04
Water	M	379.67 ± 0.09	212.11 ± 0.11

GAE: gallic acid equivalent per gram of dry weight; QE: quercetin equivalent; M: maceration; S: Soxhlet.

**Table 2:** Fatty acids composition of hexane and ethyl acetate extracts from green algae *U. lactuca*.

Fatty Acids	RT (min)	HE (%)		EAcE (%)	
		M	S	M	S
Eicosenoic acid (C20:1)	20.08	35.44 ± 0.02	6.93 ± 0.04	29.62 ± 0.07	nd
7,10-Hexadecadienoic acid (C16:2)	21.17	2.60 ± 0.05	2.93 ± 0.01	13.52 ± 0.04	nd
Palmitoleic acid (C16:1)	23.12	5.01 ± 0.07	6.37 ± 0.03	24.72 ± 0.06	nd
Palmitic acid (C16:0)	23.31	28.05 ± 0.03	46.81 ± 0.10	32.14 ± 0.08	49.92 ± 0.07
Margaric acid (C17:0)	23.87	nd	6.90 ± 0.06	nd	nd
Oleic acid (C18:1)	24.55	nd	nd	nd	28.60 ± 0.04
Linoleic acid (C18:2)	25.04	25.48 ± 0.01	23.50 ± 0.05	nd	21.48 ± 0.05
Linolenic acid (C18:3)	25.09	nd	6.56 ± 0.02	nd	nd
Stearic acid (C18:0)	25.26	3.42 ± 0.01	nd	nd	nd
SFA ^a^	31.47	31.47	53.71	32.14	49.92
UFA ^b^	68.53	68.53	46.29	67.86	50.08
UFA/SFA	2.18	2.18	0.86	2.11	1

RT: retention time; M: maceration; S: Soxhlet, HE: hexane extract; EAcE: ethyl acetate extract; nd: not detected; ^a^: saturated fatty acids (SFA); ^b^: unsaturated fatty acids (UFA).

**Table 3:** Chemical composition of ethyl acetate and methanolic extracts from green algae *U. lactuca*.

N°	Compounds	RT (min)	EAcE (%)		ME (%)	
			M	S	M	S
1	Gallic acid	15.47	nd	nd	nd	0.64
2	Catechin	18.68	1.52	8.18	nd	nd
3	4-hydroxy benzoïque	18.91	4.11	18.77	nd	3.56
4	Chlorogenic acid	19.15	1.09	4.20	11.66	7.71
5	Caffeic acid	19.45	nd	Nd	35.64	24.24
6	Syringic acid	19.74	nd	3.09	3.67	nd
7	Vanilline	23.10	3.45	nd	nd	nd
8	*p*-Coumaric acid	23.63	Nd	nd	nd	5.26
9	Sinapic acid	24.09	9.53	nd	nd	nd
10	Quercetine 3glucoside	24.52	8.07	nd	nd	nd
11	7,3′,4′-flavon-3-ol	24.92	8.31	20.68	nd	nd
12	Naringin	25.01	8.34	17.75	21.06	12.63
13	Rutin	25.16	8.29	nd	nd	nd
14	Salicylic acid	25.32	nd	15.61	14.90	11.85
15	Quercetine	25.46	8.67	nd	nd	nd
16	Cinnamic acid	25.48	8.94	nd	nd	9.66
17	Luteolin	25.64	8.91	nd	nd	9.38
18	Apigenine	25.87	7.83	nd	nd	nd
19	Kaempferol	26.1	9.11	11.69	8.10	9.11
20	Flavone	26.92	nd	nd	4.95	5.94
21	Flavanone	27.412	nd	3.79	nd	nd

RT: retention time; M: maceration; S: Soxhlet; EAcE: ethyl acetate extract; ME: methanolic extract; nd: not detected.

**Table 4:** IC_50_ values of *U. lactuca* extracts.

Extracts		IC_50_ (mg/mL)	
		DPPH	β-Carotene
EAcE	M	0.55 ± 0.02	0.47 ± 0.04
	S	0.62 ± 0.01	0.08 ± 0.14
ME	M	0.59 ± 0.13	0.41 ± 0.22
	S	0.65 ± 0.21	0.10 ± 0.05
AQE	M	0.09 ± 0.12	0.11 ± 0.17
Ascorbic Acid		0.06	-
BHA		-	0.02

EAcE: ethyl acetate extract; ME: methanolic extract; AQE: aqueous extract; M: maceration; S: Soxhlet.

**Table 5:** IC_50_ values of *U. lactuca* extracts and acarbose in α-amylase and α-glucosidase inhibition.

Inhibitors	IC_50_ (mg/mL)		
	α-Amylase		α-Glucosidase
Acarbose		0.35 ± 0.08	0.39 ± 0.04
EAcE	M	0.77 ± 0. 09 **	0.42± 0.04 ***
	S	0.78 ± 0. 16 ***	0.37± 0.05 ns
ME	M	0.81 ± 0. 05 ***	0.44± 0.06 **
	S	0.63 ± 0. 03 ns	0.27± 0.04 **
AQE	M	0.89 ± 0. 21 ***	0.51± 0.06 ***

Data are expressed as mean ± SD. ns: non-significant; significant ** *p* < 0.01; *** *p* < 0.001.

**Table 6:** The docking scores of the selected docked compounds were identified in *Ulva lactuca* extracts with the proteins (1B2Y) and (5NN8) using SP docking.

N°	Compound Name	Docking Score (kcal/mol)	
		Alpha-Amylase	Alpha-Glucosidase
1	Gallic acid	−5.298	−7.334
2	Catechin	−5.806	−5.592
3	4-hydroxy-benzoïc acid	−5.064	−5.034
4	Chlorogenic acid	−4.228	−4.589
5	Caffeic acid	−4.629	−5.425
6	Syringic acid	−4.946	−4.767
7	Vanilline	−5.272	−5.621
8	*p*-Coumaric acid	−4.223	−4.044
9	Sinapic acid	−4.304	−3.813
10	Quercetin-O-3-glucoside	−5.704	−5.218
11	7,3′,4′-flavon-3-ol	−5.923	−6.620
12	Naringin	−5.601	−5.055
13	Rutin	−6.807	−6.060
14	Salicylic acid	−5.336	−4.886
15	Quercetin	−5.187	−4.654
16	Cinnamic acid	−3.589	−3.522
17	Apigenin	−5.811	−4.766
18	Kaempferol	−5.539	−4.899
19	Flavone	−5.994	−5.319
20	Flavanone	−5.433	−5.599
21	Acarbose (standard)	−4.877	−4.925

## Data Availability

Data are contained within the article.
